# Reduced Functional Connectivity of Default Mode and Set-Maintenance Networks in Ornithine Transcarbamylase Deficiency

**DOI:** 10.1371/journal.pone.0129595

**Published:** 2015-06-11

**Authors:** Ileana Pacheco-Colón, Stuart D. Washington, Courtney Sprouse, Guy Helman, Andrea L. Gropman, John W. VanMeter

**Affiliations:** 1 Center for Functional and Molecular Imaging, Georgetown University, Washington, DC, United States of America; 2 Department of Neurology, Georgetown University Medical Center, Washington, DC, United States of America; 3 Department of Neurogenetics, Children’s National Health System, Washington, DC, United States of America; 4 George Washington University of the Health Sciences, Washington, DC, United States of America; 5 Medical Genetics Branch, NHGRI, National Institutes of Health, Bethesda, Maryland, United States of America; Peking University, CHINA

## Abstract

**Background and Purpose:**

Ornithine transcarbamylase deficiency (OTCD) is an X-chromosome linked urea cycle disorder (UCD) that causes hyperammonemic episodes leading to white matter injury and impairments in executive functioning, working memory, and motor planning. This study aims to investigate differences in functional connectivity of two resting-state networks—default mode and set-maintenance—between OTCD patients and healthy controls.

**Methods:**

Sixteen patients with partial OTCD and twenty-two control participants underwent a resting-state scan using 3T fMRI. Combining independent component analysis (ICA) and region-of-interest (ROI) analyses, we identified the nodes that comprised each network in each group, and assessed internodal connectivity.

**Results:**

Group comparisons revealed reduced functional connectivity in the default mode network (DMN) of OTCD patients, particularly between the anterior cingulate cortex/medial prefrontal cortex (ACC/mPFC) node and bilateral inferior parietal lobule (IPL), as well as between the ACC/mPFC node and the posterior cingulate cortex (PCC) node. Patients also showed reduced connectivity in the set-maintenance network, especially between right anterior insula/frontal operculum (aI/fO) node and bilateral superior frontal gyrus (SFG), as well as between the right aI/fO and ACC and between the ACC and right SFG.

**Conclusion:**

Internodal functional connectivity in the DMN and set-maintenance network is reduced in patients with partial OTCD compared to controls, most likely due to hyperammonemia-related white matter damage. Because several of the affected areas are involved in executive functioning, it is postulated that this reduced connectivity is an underlying cause of the deficits OTCD patients display in this cognitive domain.

## Introduction

Urea cycle disorders (UCD) are one of the most common groups of inborn errors of metabolism, with an estimated incidence of 1 in 30,000 births per year [1,2]. UCD can lead to an accumulation of ammonia in the blood, which then crosses the blood-brain barrier, causing a range of neurological deficits and sometimes death. Research has confirmed clinical syndromes involving deficiencies of five urea cycle enzymes and three related cofactors and transporters [3,4]. Ornithine transcarbamylase deficiency (OTCD) is an X-linked recessive disorder and the most common UCD, with an incidence of 1 in 14,000 [5]. Approximately 60% of males with OTCD present with newborn coma [6,7], while the remaining 40% are late-onset males with more peripheral mutations in the gene and a less severe phenotype [8].

The majority of children with complete OTCD have vast cognitive and motor deficits associated with neonatal hyperammonemic episodes [9,10]. Males with partial deficiencies show later disease onset and better outcome, although many still demonstrate cognitive, motor, and/or psychiatric symptoms [11]. Due to allelic heterogeneity and differential X-activation, heterozygous females show a very broad range of phenotypes. About 85% of the females are considered asymptomatic, while the remainder experience more severe symptoms such as behavioral and learning disabilities, protein intolerance, stroke-like episodes, and hyperammonemic coma [12,13]. Patients with OTCD often have trouble regulating their protein intake, which can result in episodes of hyperammonemia that cause substantial injury to the brain’s white matter [14].

Previous studies have shown that, although their IQ scores fall within the normal range, patients with partial OTCD have impairments in motor performance and nonverbal intelligence compared to controls, as shown through tasks involving fine motor skills/dexterity/speed, visual memory, attention, and math [15]. Gropman et al. (2010) also showed OTCD patients, both male and female, performed worse than controls in tests of executive functioning, such as the Stroop task and the Comprehensive Trail-Making Test (CTMT). This nonverbal learning disability is typically associated with white matter dysfunction, which is supported by a diffusion tensor imaging study that found that OTCD patients have reduced fractional anisotropy, a marker of white matter integrity, in frontal cortex as compared to controls [14].

Recently, there has been increased focus on studying the brain’s activity in a resting state [16–19]. A neural network known as the default mode network (DMN) has been consistently found in both healthy subjects and patient populations [18,19]. The DMN consists of medial prefrontal cortex (mPFC), rostral anterior cingulate cortex (ACC), posterior cingulate cortex (PCC), precuneus, and inferior parietal lobule (IPL) [16,17]. The DMN has high activity when an individual is at wakeful rest, and suppressed activity during cognitively demanding tasks [20,21]. The DMN is thought to underlie self-referential processes, such as Theory of Mind, which is defined as a person’s ability to imagine the thoughts, intentions, and feelings of others, as well as prospection, social cognition, and episodic memory [22–26].

There are also many other resting-state networks, one of which is known as the cingulo-opercular or set-maintenance network [27,28]. This network includes anterior PFC, anterior insula/frontal operculum (aI/fO), dorsal ACC, medial superior frontal cortex, and thalamus [27, 28]. It underlies executive functions such as maintenance of task rules and goals, and performance monitoring [28].

In this study, we sought to investigate for the first time differences in functional connectivity, defined as the temporal correlation between spatially remote neurophysiological events [29], of the default mode and set-maintenance networks in patients with OTCD and healthy controls. We chose to focus on these networks because: 1) they have never before been examined in OTCD patients, 2) previous studies have found abnormal connectivity in the DMN of clinical populations with symptoms akin to those of OTCD patients [30, 31], and 3) the set-maintenance network underlies many of the cognitive functions that are impaired in OTCD [28]. Based on previous findings of white matter damage and reduced integrity of frontal white matter in OTCD patients [14] as well as the nature of their cognitive deficits, we hypothesized that OTCD patients would demonstrate reduced functional connectivity as compared to controls, particularly in frontal regions.

## Methods

### Participants

All participants were recruited as part of an NIH-funded Rare Diseases Clinical Research Center established to study the natural history of UCD. OTCD patients were recruited through the Online Rare Diseases Clinical Research Network (RDCRN) registry, the Society for Inherited Metabolic Disease (SIMD), and colleagues of the principal investigator known to serve OTCD patients in metabolic clinics throughout the country. Control subjects were recruited through advertisements approved by the Institutional Review Board and posted throughout Georgetown University Hospital, Medical Center, and graduate school.

Sixteen participants with partial OTCD (13 females, 3 males) and 22 control subjects (16 females, 6 males) without UCD or neurological symptoms participated in this study (Table 1). Patients ranged in age from 6 to 59 years (mean 30 years), while control subjects ranged in age from 8 to 55 years (mean 27 years). Patients included males with late-onset OTCD as well as both symptomatic and asymptomatic heterozygous females, with varying ages of diagnosis and degree of metabolic control. Participants were not taking psychotropic medication at the time of the study, nor did they receive any drug treatment as part of the study. All participants had IQ scores above 70 as measured by the Wechsler Abbreviated Scales of Intelligence (WASI). The two groups did not differ significantly in age, or IQ scores (Table 1). Neither patients nor healthy volunteers had a history of neurological (other than OTCD-related issues) or psychological conditions. Claustrophobia and metal implants were part of the exclusionary criteria. Due to excessive head motion, the data for 3 OTCD patients was excluded from the final analyses leaving 13 subjects in the OTCD group (11 F, 2 M; ages 6 to 59, mean 34 years). After these exclusions, the two groups did not differ significantly in motion (Table 1).

**Table 1:** Participant Demographics.

Subjects (*N* = 38)	Controls (*N =* 22)	OTCD (*N* = 16)
Age	26.68 ± 15.42	29.69 ± 20.30
Sex	16 F, 6 M	13 F, 3 M
Full-scale IQ	110.9 ± 16.29	104.86 ± 15.51
Performance IQ	107.85 ± 17.79	107.36 ± 14.04
Verbal IQ	111.4 ± 12.57	101.43 ± 17.57
Framewise Displacement (mm)	0.17 ± 0.09	0.20 ± 0.06✦✦

✦✦These values reflect the framewise displacement of patients after the exclusion of 3 OTCD patients due to excessive head motion (*N* = 13).

### Semi-Structured Clinical Interview

Both controls and OTCD patients participated in a semi-structured clinical interview with a neurologist (coauthor: ALG) and a neurological assessment, which in the controls was used to rule out any unknown neurological disorders. A semi-structured interview typically has a framework of themes to be explored, leaving room for new questions to be asked based on the subjects’ responses. In this study, questions concerned the onset, presence, and severity of symptoms related to OTCD, as well as participants’ medical histories. Based on their answers to these questions, OTCD patients were also assigned severity scores based on the scale developed by Msall et al. (1988) [32].

### Data Acquisition

All scanning was performed on a 3T Siemens Tim Trio MRI scanner (Erlengen, Germany) at the Center for Functional and Molecular Imaging at Georgetown University Medical Center. We minimized head movement using padding that was fitted to hold the subject’s head firmly and comfortably in the coil. Resting-state BOLD images (TR = /TE = 2000 ms/30 ms, 90° flip angle, 256 mm FOV, 64 x 64 matrix, 36 slices, 3.7 mm slice thickness with 0.3 mm gap for an effective resolution of 4.0 mm^3^, 5:16 total scan time) were collected to examine the DMN and other resting-state networks. For this scan, participants were instructed to keep their eyes closed without falling asleep, and to relax and let their mind wander. High-resolution anatomical images were acquired using a T1-weighted MPRAGE sequence (TR/TE = 1900 ms/2.52 ms, 9° flip angle, 256 mm FOV, 256 x 256 matrix, 160 slices for an effective resolution of 1.0 mm^3^).

### Ethics Statement

The Children’s National Medical Center Institutional Review Board (FWA00004487) approved this study (Study #: Pro00000420; Continuing Review #: CR00002192), including all of the methods used to obtain these data, methods of consent, and study protocols. We obtained written informed consent from all adults, as well as from the parents of the minors enrolled in the study. Minors also provided written assent.

### Image Analysis

SPM8 software (http://www.fil.ion.ucl.ac.uk/spm/software/spm8/) was used to preprocess the resting-state images. The first 3 volumes, or whole-brain 3D images, were excluded to allow magnetization to reach equilibrium. Each subject’s images were corrected for slice timing, realigned to correct for interscan head movement, and co-registered to the subject’s anatomical scan. Anatomical images were segmented using SPM8’s segmentation module, the output of which was used to normalize the subject’s data into Montreal Neurological Institute (MNI) space using an affine transformation, very light regularization and trilinear interpolation. Finally, images were smoothed with an 8 mm Gaussian kernel.

We then assessed and corrected for motion using the scrubbing method described in Power et al. (2012) [33]. First, we identified and removed every volume with more than 0.5 mm of translation for each subject. At this point, 3 subjects were excluded from further analyses due to excessive head motion, defined for our purposes as more than 32 volumes with excessive motion or less than 4 minutes of usable scan time. At the end of this process, subjects were left with varying numbers of volumes. Of the remaining subjects, the one with the most motion (22 volumes with excessive motion) still had enough volumes to allow for the assessment of resting state functional connectivity (130 volumes, 4:20 total scan time). In order to standardize the number of volumes across subjects, thus avoiding biasing the results towards the subjects who were left with more volumes, we used a random number generator to delete random volumes from every other subject, such that all subjects had a total of 130 usable volumes.

Using the same analysis procedures as we have done in our investigations of differences in functional connectivity between autistic and typically developing children [34], we employed a two-stage analytic approach. We first used an independent component analysis (ICA) to identify the nodes of each network in a data-driven manner. This is followed by a region of interest (ROI) analysis to assess differences between the subject groups with respect to the connectivity within the network as a whole using a two-way ANOVA followed by post-hoc one-way ANOVAs to determine which pairs of ROI led to the group differences. The details of these procedures are given below.

### Independent Component Analysis (ICA)

ICA is a model-free approach that separates a set of signals into independent, uncorrelated, and non-Gaussian components [35]. Here, we used ICA to identify the fluctuations in BOLD signal associated with the default mode and set-maintenance networks. Using FSL MELODIC (http://www.fmrib.ox.ac.uk/fsl/m-elodic2/index.html), we selected “multi-session temporal concatenation” to perform separate group ICAs on the resting-state images from our OTCD patient group and our normal control group. We did not impose a predetermined number of components, thus allowing MELODIC to automatically estimate the number of components generated for each group. We then visually identified the components that corresponded to the DMN and the set-maintenance networks by comparing each component to previously published maps of the DMN and set-maintenance network, respectively [16,17,27,28].

### ROI Analyses

In order to directly compare differences in functional connectivity between specific nodes of each network between our patient and control groups, we conducted temporal correlations of the signal time courses within specific regions of interest (ROIs). We used MRIcron (http://www.nitric.org/projects/mricron) to identify ROIs corresponding to nodes of each network in the control group, based on a threshold of 87.5% of ICA map maximum. The ROIs were constructed from these outlined regions using the MarsBar toolkit (http://www.mrccbu.cam.ac.uk/Imaging/m-arsbar.html).

For each ROI within each network, the residual time courses for each voxel (a 3D pixel) in an ROI were averaged, and the mean residual time courses within each pair of ROIs were partially correlated to assess functional connectivity. For each subject, this analysis generated a correlation value, which reflected the strength of functional connectivity between each ROI pair. In order to minimize the effect of physiological factors (such as respiration or heart rate) common to the time-courses of all ROIs, we linearly regressed out the average signal time-course from non-neuronal sources, specifically from cerebral spinal fluid (CSF) and white matter [36].The correlation *r*-values were normalized using Fisher’s *r-*to-*Z*-transform:

$$z\;\equiv \;\text{arctanh}\left(r\right)=\frac{1}{2}\;\text{ln}\;\left(\frac{1+r}{1-r}\right)$$

where *r* is the Pearson’s Product Moment Correlation and *z* is the Fisher’s *z*.

Additionally, in order to ensure that any observable between-group differences were not due to the imposition of the control group ROIs on the patient group, we conducted a separate set of ROI analyses using neutral predetermined ROIs outlined in Shirer et al. (2013) [37].

## Results

### Clinical Observations

Both the patients and the control subjects participated in a semi-structured interview and underwent neurological assessment by a neurologist. Typically affected cognitive domains in the patients included non-verbal learning, fine motor processing, reaction time, visual memory, attention, and executive function. Based on the interview, the patients were found to suffer from high levels of anxiety as a result of becoming easily overwhelmed when carrying out complex tasks, such as driving.

Patients were rated 0–5 on a severity scale based on their medical history and symptomatology [32]. The breakdown of our patients’ severity scores, as well as what each score represents, can be observed in Table 2. Because our subjects were all high-functioning patients with IQ scores over 70, we had no patients with severity scores of 3 or higher.

**Table 2:** OTCD Patient Severity Scores.

Severity Score	Score Meaning	Number of OTC Patients
0	Asymptomatic	4
1	Recurrent hyperammonemia manifested by vomiting and lethargy	5
2	Single episode of Stage III coma without increased intracranial pressure	7
3	Multiple episodes of Stage III coma without increased intracranial pressure	0
4	Multiple episodes of Stage III-IV coma with a single episode of increased intracranial pressure	0
5	Multiple episodes of Stage III-IV coma with multiple episodes of increased intracranial pressure	0

### DMN

**Fig 1:**
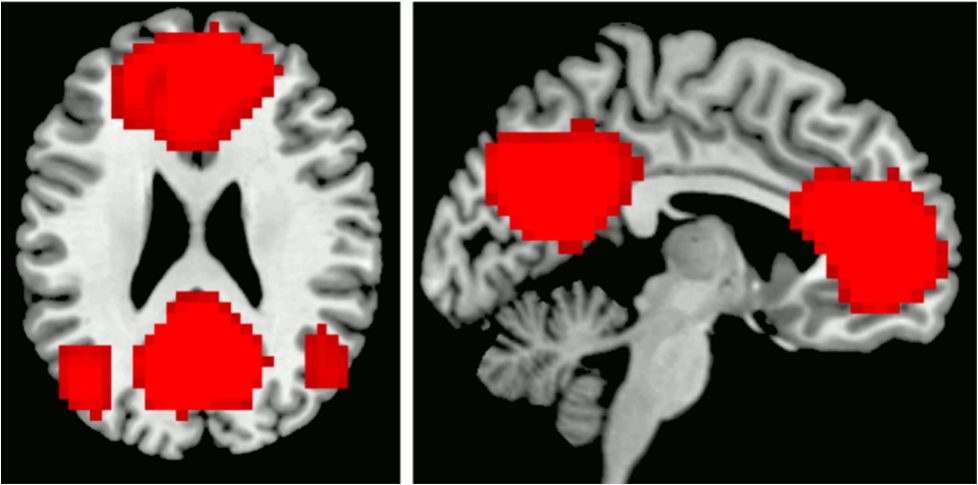
DMN. The default mode network of both subject groups is composed of an ACC/mPFC node, a PCC/precuneus node, and bilateral IPL nodes.

The group ICA generated a total of 26 components for control subjects and 36 components for OTCD patients. We identified the DMN, consisting of the ACC/mPFC node, the precuneus/PCC node, and bilateral inferior parietal lobule (IPL) nodes, in both subject groups (Fig 1).

**Fig 2:**
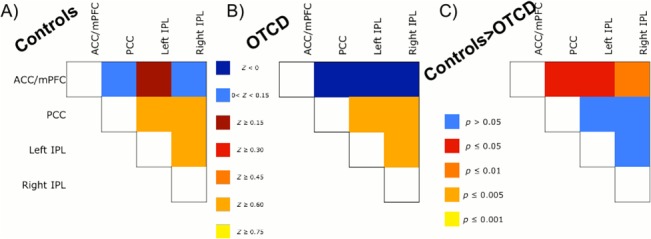
ROI Analyses Results. A) Z-scores reflecting degree of functional connectivity between pairs of ROIs in the Control group. B) Z-scores reflecting degree of functional connectivity between pairs of ROIs in OTCD patient group. C) Controls have significantly greater DMN functional connectivity than OTCD patients between ACC/mPFC node and bilateral IPL.

The pair-wise correlation matrices obtained using the ROI-analysis are shown in Fig 2A and 2B. There were a total of 6 ROI pairs, and all ROIs were based on the DMN nodes from the control group’s ICA.

We then tested for differences in internodal connectivity between groups, using a 2 (Group) x 6 (ROI pair) ANOVA, with the z-scores reflecting functional connectivity as the dependent variable and age as a covariate. There was a main effect of group, showing that control subjects had overall greater functional connectivity in DMN nodes than OTCD patients, *F* (1, 197) = 5.46, *p* = 0.02, ƞ^2^ = 0.017. There was also a main effect of ROI pair, showing that there was greater functional connectivity between some ROI pairs than others, *F* (5, 197) = 23.75, *p*<0.001, ƞ^2^ = 0.37. There was no significant interaction between group and ROI pair (*F* [5, 197] = 1.132, *p* = 0.35), which indicates that the differences in functional connectivity between groups held for all ROI pairs, though it is possible that this lack of interaction stems from the small size of our sample. The covariate, age, was not significantly related to the degree of functional connectivity, though it did approach significance (*F*[1, 197] = 0.38, *p* = 0.054). In order to identify the ROI pairs that accounted for the between-group difference, we ran a series of post-hoc one-way ANOVAs. After controlling for age, controls had greater connectivity than patients between the ACC/mPFC node and bilateral IPL nodes, *F*(1, 32) = 5.62, *p* = 0.024, ƞ^2^ = 0.12 (left), and *F*(1,32) = 7.95, *p* = 0.008, ƞ^2^ = 0.19 (right), as well as between the ACC/mPFC node and the PCC node, *F*(1, 32) = 4.61, *p* = 0.04, ƞ^2^ = 0.20, as shown in Fig 2C. The group difference in connectivity between ACC/mPFC and right IPL remained significant after a Bonferroni correction for multiple comparisons (α = 0.05, *n* = 6). We obtained the same results when conducting the analyses with the neutral ROIs, which are discussed in greater detail in S1 File.

We also examined the relationship between internodal connectivity of the patients’ DMN and their symptoms by correlating the severity scores with the *z*-scores of the node pairs, but found no significant correlations.

### Set-Maintenance Network

**Fig 3:**
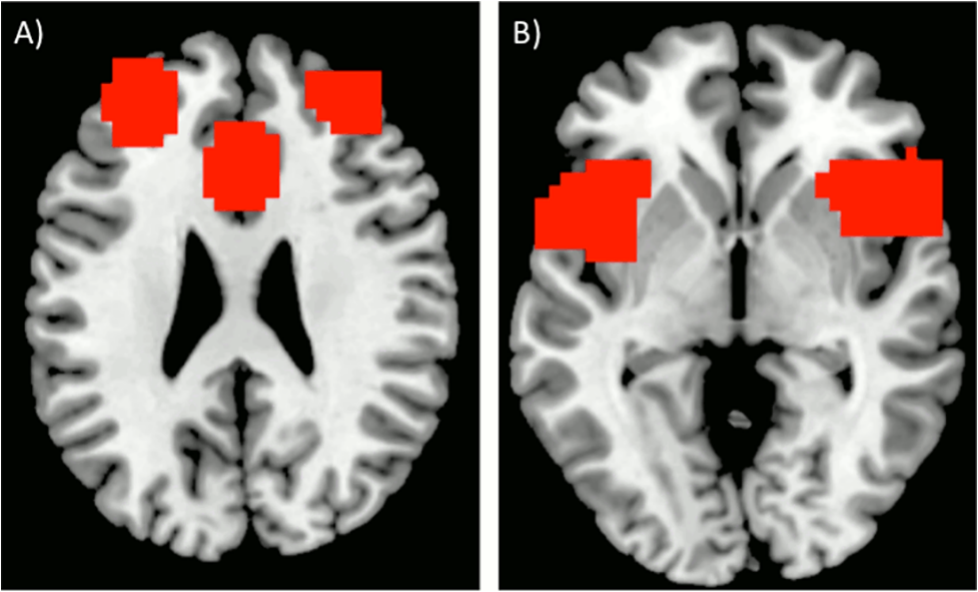
Set-Maintenance Network. The set-maintenance network is composed of A) ACC and bilateral SFG, and B) bilateral aI/fO.

**Fig 4:**
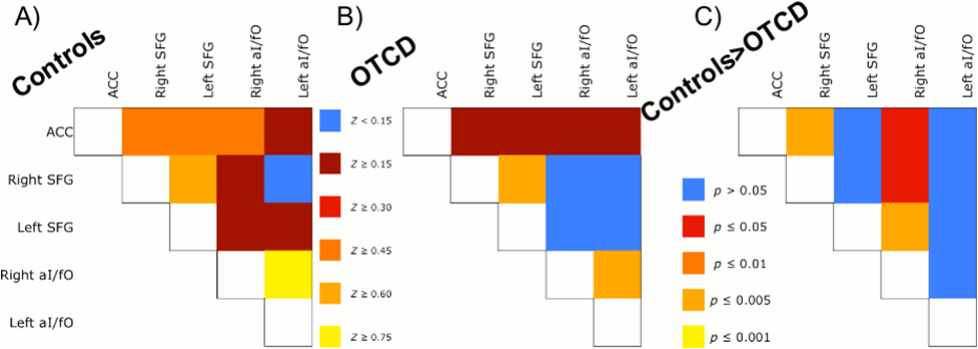
ROI Analyses Results. A) Z-scores reflecting degree of functional connectivity between pairs of ROIs in the Control group. B) Z-scores reflecting degree of functional connectivity between pairs of ROIs in OTCD patient group. C) Controls have significantly greater set-maintenance functional connectivity than OTCD patients between ACC node and bilateral SFG.

The set-maintenance network appeared within a single component for control groups, and within two components for the patient group. It consisted of an ACC node, bilateral superior frontal gyrus (SFG) nodes, and bilateral aI/fO nodes (Fig 3), though the right aI/fO node was missing in the patient group. The control group’s ICA component also included bilateral IPL nodes. However, these were excluded from our analyses, as they are not typically thought to be a part of the set-maintenance network [27,28]. To further investigate the degree of functional connectivity, we repeated the methods described above. There were a total of 10 ROI pairs, and all ROIs were based on the nodes from the control group’s ICA (Fig 4A and 4B).

We then tested for differences in internodal connectivity between groups with a 2(Group) x 10 (ROI pair) ANOVA, using age as a covariate. There was a main effect of age, *F* (1, 329) = 6.76, *p* = 0.01, ƞ^2^ = 0.013, showing that the age of participants influenced their degree of functional connectivity. Even after age-related variance was accounted for, there was a main effect of group, *F*(1, 329) = 18.95, *p*<0.001, ƞ^2^ = 0.037, suggesting reduced functional connectivity in set-maintenance network nodes across all ROI pairs, as well as a main effect of ROI pair, *F*(9, 329) = 16.01, *p*<0.001, ƞ^2^ = 0.28, indicating that some ROI pairs have greater functional connectivity than others. There was no interaction between Group and ROI Pair (*F* [9, 197] = 0.67, *p* = 0.74), suggesting that the differences in functional connectivity across set-maintenance nodes were largely ubiquitous. A series of post-hoc one-way ANOVAs were used to identify the specific ROI pairs that accounted for the Group differences while controlling for age, controls had greater internodal connectivity between the ACC node and the right SFG node, *F*(1, 32) = 9.37, *p* = 0.005, ƞ^2^ = 0.22, as well as between the ACC and the right aI/fO node, *F*(1, 32) = 4.61, *p* = 0.039, ƞ^2^ = 0.12, between the right aI/fO and the bilateral SFG, *F*(1, 32) = 10.43, *p* = 0.003, ƞ^2^ = 0.25 (left) and *F*(1, 32) = 5.68, *p* = 0.023, ƞ^2^ = 0.15 (right) (Fig 4C). Of these 10 comparisons, the group difference in connectivity between ACC and right SFG as well as the connectivity between left SFG and right aI/fO remained significant after applying a Bonferroni correction for multiple comparisons (α = 0.05, *n* = 10).

The results of the analyses involving the neutral ROIs validated the aforementioned results in that 3 of the 4 ROI pairs were also significant using these ROIs. The difference in connectivity between the right aI/fO node and right SFG only approached significance, *F*(1, 32) = 3.98, *p* = 0.055. Further, the controls also showed greater connectivity between the ACC and left SFG nodes, *F*(1, 32) = 7.22, *p* = 0.011. These results are discussed in full detail in S2 File.

As with the DMN, we examined the relationship between internodal connectivity of the patients’ set-maintenance network and their symptoms. However, we found no significant correlations between the patients’ severity scores and their functional connectivity.

## Discussion

In this study, we found significant differences in DMN functional connectivity in individuals with partial OTCD as compared to controls. Our results indicate that OTCD subjects had reduced overall functional connectivity between nodes of the DMN, particularly between the ACC/mPFC node and bilateral IPL nodes and between the ACC and PCC/precuneus node, likely reflecting damage to white matter tracts caused by hyperammonemic episodes.

The DMN is a network thought to be associated with Theory of Mind, or the ability to imagine the thoughts, intentions, and feelings of others, as well as self-reflection, prospection, social cognition, and episodic memory [22–26]. The mPFC and more lateral DMN structures such as the IPL seem to be involved in Theory of Mind reasoning [25, 26], while the PCC is thought to be involved in internally-directed cognition [38]. To the best of our knowledge, no assessments of Theory of Mind have been performed on OTCD populations. It would be interesting to investigate whether the impaired connectivity observed for OTCD patients in this study is reflected in deficits in their ability to engage in Theory of Mind reasoning. Deficits of this nature would have implications for the social functioning of OTCD patients.

The set-maintenance network, on the other hand, is active both at rest and during task performance, providing stable set-maintenance throughout a task period [28]. Our study also revealed significantly reduced functional connectivity in this network in the OTCD patients relative to the control subjects, particularly between right aI/fO node and bilateral SFG nodes, between the right aI/fO and ACC node, and between the ACC node and right SFG.

The anterior insula is thought to play a role in domain-general attentional control [27], as well as in goal-oriented tasks and performance monitoring [39]. Similarly, activity in dorsal ACC is associated with attentional control and working memory [40–43], often increasing with increased effort, complexity, or attention [41, 44]. It has also been associated with error detection and response correction [45]. SFG activity has also been implicated in aspects of executive functioning such as working memory and task switching [46,47]. OTCD patients have been known to display deficits in these executive aspects of cognition, especially as tasks become more challenging [9, 48]. It is plausible that some of these deficits could be related to the impaired connectivity surrounding the aI/fO, the ACC, and SFG, all of which underlie executive functioning. The patients’ reports of feeling anxious and overwhelmed when performing complex tasks are likely related to the reduced connectivity in the set-maintenance network.

Previous studies on OTCD populations found increased activation in frontal regions, including the ACC, relative to controls despite a lack of differences in task performance, suggesting a model of prefrontal inefficiency [49]. It is possible that this increased ACC activity is a mechanism employed to compensate for the poor connectivity between the ACC and other areas critical for executive functioning, such as the insula, IPL, and SFG, as demonstrated by the current study.

Though we did not find any correlations between symptom severity and reduced functional connectivity in either of these networks, it is important to note that the severity scale used in this study is outdated, as it only takes into account patients who present with clinically obvious symptoms. Some of the “asymptomatic” OTCD carriers have mild symptoms, while others show no symptoms yet have MRS findings of elevated glutamine and low myoinositol [50], as well as some cognitive deficits [48]. Therefore, we feel that this scale does not accurately reflect the subtle differences that exist within OTCD patients with regards to symptom severity and cognitive functioning. We believe that the absence of this correlation does not mean that the correlation does not exist, but rather that a better, more specific scale needs to be developed before we can reach a definitive conclusion.

Overall, our findings provide preliminary evidence of reduced functional connectivity in OTCD, likely caused by hyperammonemia-related white matter injury. These results support previous findings of frontal white matter damage and deficits of executive functioning [9,14,49]. It must be noted that although we found significant differences between OTCD patients and control subjects, our findings are limited by both the small size and heterogeneity of our sample, especially the wide age range of our participants. Due to the rarity of the disorder at hand and the dearth of research including children with partial OTCD, we were unable to create larger, more age-specific patient groups. For similar reasons and lack of statistical power, we were also unable to separate the OTCD patient group into symptomatic and asymptomatic groups. Additionally, it is plausible that results were affected by unknown effects of OTCD on the variability of hemodynamic response function (HRF) though it is unlikely that such differences in the HRF would affect functional connectivity. Finally, the relationship between functional and structural connectivity is not always a straightforward one [51]. While some multimodal imaging studies have shown correspondence between functional and structural connectivity [52], others have found that the relationship between the two may depend on other factors, such as age and task goals [53–55]. Thus, further investigation using other imaging modalities, especially DTI, is needed to confirm that the findings of reduced functional connectivity discussed in the current study are in fact due to reduced structural integrity of the white matter of OTCD patients.

## Supporting Information

S1 File DMN ROI Analyses Results Using Neutral ROIs. (DOCX)

S2 File Set-Maintenance Network ROI Analyses Results Using Neutral ROIs. (DOCX)
