# Supplementary material for: Reduced Functional Connectivity of Default Mode and Set-Maintenance Networks in Ornithine Transcarbamylase Deficiency
Source: PLoS One. 2015 Jun 11;10(6):e0129595. doi: 10.1371/journal.pone.0129595 (PMC4466251; doi:10.1371/journal.pone.0129595)
Supplement: S1 File — (DOCX) [file pone.0129595.s001.docx]

**Text S1. Results of ROI Analysis using neutral DMN ROIs**

There were a total of 6 ROI pairs and all ROIs were selected from the networks established in Shirer et al. (2013) ^27^. We tested for differences in connectivity between groups using a 2 (Group) x 6 (ROI pair) ANOVA with age as a covariate. There was a significant main effect of age, *F*(1, 197)=9.38, *p*=0.003. There was also a main effect of group, such that controls showed greater overall functional connectivity between DMN nodes, *F*(1, 197)=25.38, *p*=0.00, and a main effect of ROI, *F*(1, 197)=8.80, *p*=0.00. There was no Group by ROI interaction.

These results were followed by post-hoc one-way ANOVAs to identify the nodes responsible for the main effect of group. After controlling for age, control subjects showed greater connectivity than OTCD patients between the ACC/mPFC and bilateral IPL nodes, *F*(1, 32)=14.60, *p*=0.001 (left) and *F*(1, 32)=6.42, *p*=0.016 (right), as well as between the ACC/mPFC and the PCC/precuneus node, *F*(1, 32)=8.79, *p*=0.006.
