# Supplementary material for: Reduced Functional Connectivity of Default Mode and Set-Maintenance Networks in Ornithine Transcarbamylase Deficiency
Source: PLoS One. 2015 Jun 11;10(6):e0129595. doi: 10.1371/journal.pone.0129595 (PMC4466251; doi:10.1371/journal.pone.0129595)
Supplement: S2 File — (DOCX) [file pone.0129595.s002.docx]

**Text S2. Results of ROI Analysis using neutral set-maintenance ROIs**

There were a total of 10 ROI pairs and all ROIs were selected from the networks established in Shirer et al (2013) ^27^. We tested for differences in internodal connectivity in the set-maintenance network between groups using a 2 (Group) x 10 (ROI pair) ANOVA, using age as a covariate. The covariate, age, had a significant effect on the degree of connectivity, *F*(1, 329)=4.61, *p*=0.033. After controlling for age, there was a main effect of group, *F*(1, 329)=23.43, *p*=0.00, suggesting controls had greater overall functional connectivity between set-maintenance nodes, and a main effect of ROI, *F*(1, 329)=14.22, *p*=0.00, suggesting greater connectivity between some ROI pairs than others. There was no significant interaction.

These analyses were followed up with post-hoc one-way ANOVAs using age as a covariate, which aimed to identify the ROI pairs causing the main effect of group. Controls displayed greater connectivity than OTCD patients between the ACC and bilateral SFG nodes, *F*(1, 32)=7.22, *p*=0.011 (left) and *F*(1, 32)=4.70, *p*=0.038 (right), as well as between the ACC node and the right aI/fO node, *F*(1, 32)=5.70, *p*=0.023. Finally, controls also showed greater connectivity between the right aI/fO node and the left SFG, *F*(1, 32)=7.20, *p*=0.011, while the group difference in connectivity between right aI/fO and the right SFG only approached significance, *F*(1, 32)= 3.98, *p*=0.055.
